# *Lactobacillus rhamnosus* GG for Cow's Milk Allergy in Children: A Systematic Review and Meta-Analysis

**DOI:** 10.3389/fped.2021.727127

**Published:** 2021-10-22

**Authors:** Weifu Tan, Zhicong Zhou, Wei Li, Han Lu, Zemin Qiu

**Affiliations:** ^1^Department of Pediatrics, Dongguan Binhaiwan Central Hospital, Jinan University, Dongguan, China; ^2^Department of Pediatrics, The First Affiliated Hospital, Jinan University, Guangzhou, China; ^3^Department of Obstotrics and Gynocology, The First Affiliated Hospital, Jinan University, Guangzhou, China

**Keywords:** cow's milk allergy, *Lactobacillus rhamnosus* GG, probiotics, systematic review, meta-analysis

## Abstract

**Objective:** Cow's milk allergy (CMA) is a common allergic disease. Probiotics have been suggested as a treatment for CMA, with *Lactobacillus rhamnosus* GG (LGG) being one of the important predominant choices. Despite reports on this topic, the effectiveness of application in CMA remains to be firmly established.

**Methods:** To assess the effects of LGG on CMA in children, the PubMed/Medline, Embase, Cochrane Library, and Web of Science databases were searched for studies on LGG in treatment of CMA, which were published in the English language.

**Results:** Ten studies were finally included. Significantly higher tolerability rates favoring LGG over controls were observed [risk ratio (RR), 2.22; 95% confidence interval (CI), 1.86–2.66; *I*^2^ = 0.00; moderate-quality evidence]. There were no significant differences in SCORAD values favoring LGG over the placebo (mean difference, 1.41; 95% CI, −4.99–7.82; *p* = 0.67; very low-quality evidence), and LGG may have improved fecal occult blood (risk ratio, 0.36; 95% CI, 0.14–0.92; *p* = 0.03; low-quality evidence).

**Conclusion:** We found that LGG may have moderate-quality evidence to promote oral tolerance in children with CMA and may facilitate recovery from intestinal symptoms. However, this finding must be treated with caution, and more gpowerful RCTs are needed to evaluate the most effective dose and treatment time for children with CMA.

**Registration number:** CRD42021237221.

## Introduction

Food allergy is common in children and has an increasing prevalence worldwide. It increased significantly from 3.5% in 1999 to 7.7% in 2009 in Chinese children (1) and from 3.4% in 1997–1999 to 5.1% in 2009–2011 in American young children (2). Cow's milk allergy (CMA) is one of the most common immune-mediated allergic diseases in children (3). However, most allergic infants can spontaneously acquire milk tolerance before the age of three (4).

The mechanism of CMA has not been fully elucidated. It may be related to cellular immunity and humoral immunity. Based on the expressions of the serum-specific immunoglobulin E (IgE), CMA can be divided into three types: IgE-associated, non-IgE-associated, and mixed (IgE and non-IgE) CMA (5–8). IgE-specific antibodies produced by B cells were observed in blood samples of CMA children. Additionally, T cells that are active on various milk proteins (whey protein and casein) can be extracted from the blood samples of these patients. When a specific T cell is activated, the cytokine profile of the T cell will affect the subsequent B cell response, leading to the production of immunoglobulins by the B cell. This indicates that the production of immunoglobulin is mediated by T cell cytokines. Thus, the patients' exposure to cow's milk protein causes a T cell reaction and allergies or sensitization (4). Simultaneously, the IgE-specific antibodies produced by B cells also binds to mast cells, leading to the degranulation of mast cells and the release of histamine, eventually causing allergic symptoms. Therefore, IgE-associated CMA may cause the following symptoms: (i) skin symptoms, including urticaria and blisters; (ii) angioedema; (iii) throat edema; (iv) respiratory symptoms, including dyspnea, coughing, and wheezing attacks; (v) gastrointestinal symptoms, including oral itching, abdominal pain, vomiting, diarrhea, and blood in the stool; and (vi) circulatory symptoms, including dizziness, confusion, hypotension, and shock (6, 9). Unlike IgE-mediated rapid allergic reactions, non-IgE-mediated reactions are usually delayed 2 h after ingestion (10). Children with non-IgE-mediated CMA may have the following specific diseases or symptoms: gastrointestinal disorders, including protein-losing enteropathy, dietary protein enterocolitis/proctitis/proctocolitis, colic, constipation, and respiratory disorders, such as pulmonary hemosiderosis (i.e., Heiner syndrome) (11, 12).

Current treatments for CMA are limited. Recently, the role of aberrant gut flora in infant CMA has triggered extensive research. The gut microbiota may influence the future outcome of children's food allergies in the early stages of infancy (13). Therefore, probiotics are also used in the management of CMA. The results of an animal experiment show that Lactobacillus strains can be used as an effective tool to treat food allergies by regulating immunity and intestinal microbiota (14).

Probiotics are a group of oral or biological organisms that have potential health benefits (15). The therapeutic mechanism of probiotics on CMA intestinal symptoms has always been the focus of research. According to the existing research results, it may include the following mechanisms: (i) exclusion or inhibition of pathogen damage through the direct action of strains or by affecting other symbiotic flora in the intestine (16); (ii) regulation of signal transduction pathways (such as NF-κ B, Akt, and MAPK-dependent pathways) which enhance the ability of epithelial barrier function, which can cause mucus secretion or enhance cell tight junctions (17, 18); and (iii) regulation of the host immune response exertion strain-specific local and systemic effects (19).

LGG was first identified in healthy adult fecal samples by Sherwood Gorbach and Barry Goldwin and was patented in 1989. Because of its resistance to acid or bile and its stickiness to the intestinal epithelium, it was considered a potential probiotic strain (20). The results of *in vitro* experiments showed that supplementing infant formula with probiotics (LGG) may provide additional benefits (21). Some trials reported LGG to be beneficial in the treatment of CMA (22, 23). However, a systematic review of the Cochrane Library included six studies with a total of 2,080 infants, evaluating the results of the use of probiotics in allergic diseases and/or food allergies. The results of the review showed that all studies reporting significant benefits used probiotic supplements containing LGG (24). The authors concluded that there is insufficient evidence to recommend the addition of probiotics to baby food to prevent allergic diseases or food allergies. It is worth noting that the results of this review should be treated with caution because of the high rate of patient loss to follow-up (17–61%). Considering the above, we believe that it is important to use scientific and rigorous systematic reviews and meta-analysis methods to evaluate the latest evidence of LGG in the CMA field for both effectiveness and safety aspects. This review aimed to investigate the efficacy and safety of LGG in the treatment of allergic symptoms in CMA children under 3 years of age.

## Method

### Criteria for Review

#### Study Types

Due to the small number of clinical studies and sample sizes regarding the use LGG treatment on CMA, we introduced randomized and quasi-randomized control trials in this review to scientifically expand the number of samples included in the study. The language of these selected studies should be English only.

#### Types of Participants

The inclusion criteria were (1) children ≤ 3 years old, who have been diagnosed with CMA in accordance with the guidelines of CMA (25) and whose allergic symptoms were rated by an authoritative pediatrician using the severity score of atopic dermatitis: (SCORing of Atopic Dermatitis [SCORAD] index (26); (2) the experimental group was only administered LGG probiotics, while the control group received a placebo, non-LGG probiotics, or mixed without LGG; (3) CMA symptoms were IgE-mediated, non-IgE-mediated, or both (mixed type); and (4) the results included changes in CMA symptoms, milk tolerance, and blood and fecal cytokine levels. All results are expressed as mean ± standard deviation (SD), rate, or a specific number of patients. After avoiding the diet for 2–4 weeks, the children were subjected to an open challenge. Subsequently, if symptoms of the CMA were observed, the diagnosis of CMA would be confirmed, and the child would be included.

The tolerance rate of milk allergy was calculated based on the number of people who achieved tolerance at the end of the follow-up. We also chose the SCORAD index to evaluate the improvement of allergic symptoms. The SCORAD index is a tool developed by the European Task Force on AD in 1993 (26) to assess the severity of atopic dermatitis and improve its management. Doctors also use it to assess the severity of food allergy symptoms. The SCORAD index is based on the (A) degree of disease, (B) intensity of the six main allergic symptoms, and (C) subjective symptoms. The total score is expressed by the formula A/5 + 7B/2 + C. Moreover, some cytokines or components in blood and fecal samples can often reflect the degree of intestinal inflammatory response. In this study, tumor necrosis factor-α (TNF-α), α-antitrypsin (AT), eosinophil cationic protein (ECP), and fecal occult blood were selected as markers to evaluate intestinal inflammatory injury. Studies that did not qualify the inclusion criteria were excluded.

#### Literature Search

Using a standard search with explicit inclusion and exclusion criteria, two authors (WT and HL) evaluated full articles classified as “unclear” or “include.” Another author (WL) resolved disagreements through discussion and using the guidelines published by the *Cochrane Handbook for Systematic Reviews of Interventions* (27).

An exhaustive search was carried out using the PubMed, Embase, and Cochrane Library databases. We also set a filter for studies (language: English; subjects: human; published before: March 1, 2021). To have a comprehensive search, we used PubMed MeSH words and free text words to form a search string by combining the most appropriate Boolean operators. This search string combined all the words related to milk allergy, *L. rhamnosus* GG, and children. We have expanded the scope of the search literature and looked for additional references in SCOPUS (http://www.scopus.com/), ISI Web of Science (http://apps.webofknowledge.com/), ProQuest Dissertation & These Database (https://proquest.libguides.com/), and Open SIGLE (accessed at Opensigle.inist.fr). We also searched several clinical research registry databases, such as the Chinese Clinical Trial Register, Australian New Zealand Clinical Trials Registry, The Netherlands National Trial Register, and ClinicalTrials.gov.

In order to search in the mentioned databases, the following search strategies were used: (*L. rhamnosus* GG or Culturalle or Lactobacillus GG or probiotic) AND (Milk Hypersensitivity or Hypersensitives, Milk or Milk, Hyersensitivies or Allergies, Milk or Milk Allergies or Allergy, Milk or Milk/adverse effect or cow's milk allergy^*^ or cow's milk protein allergy^*^) AND (infant, newborn or newborn or neonate or neonatal or child or children or Newborn or infan^*^ or neonat^*^) AND (human not animal) AND (randomized controlled trial or controlled clinical trial or randomized or placebo or clinical trials as topic or randomly or trial or clinical trial).

**Figure 1:**
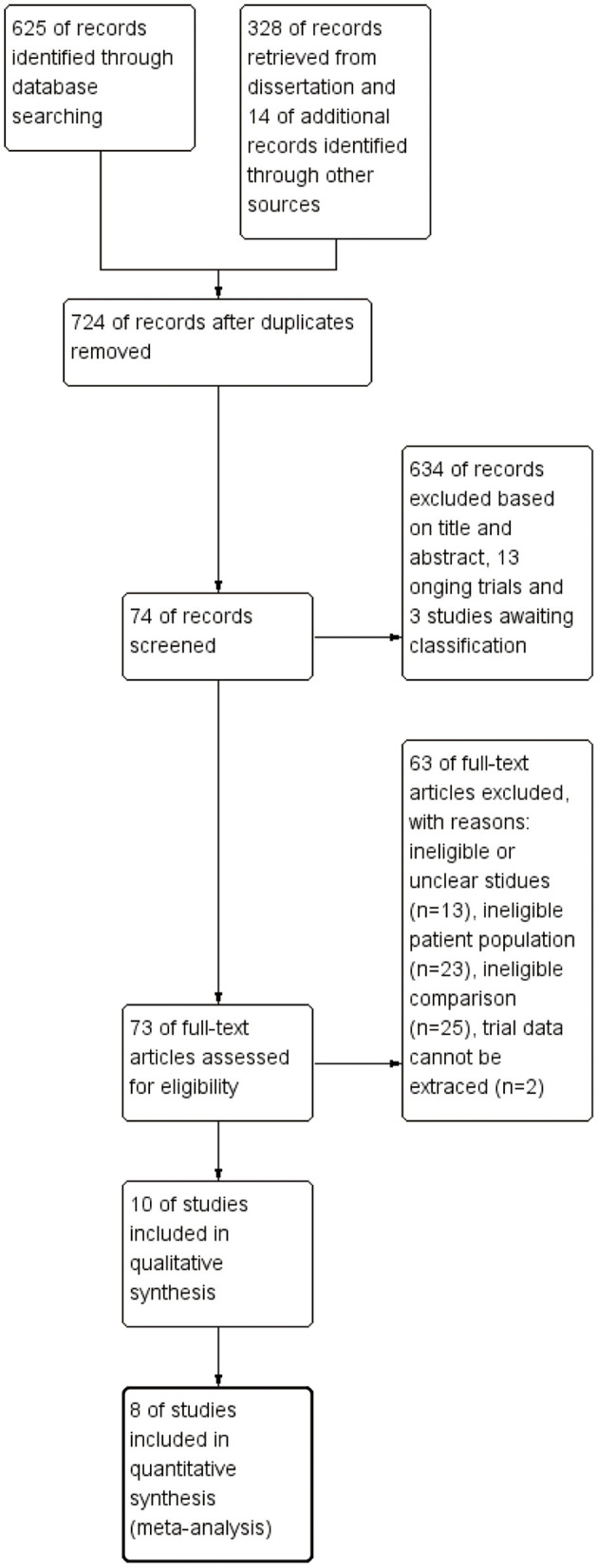
Search results flowchart. The number of related papers at each point is given.

Furthermore, we searched the list of references in each research report to collect more information. All works were in English. Furthermore, ongoing research, conference abstracts, or lack of detailed data were also excluded. The detailed literature screening process is shown in Figure 1.

#### Data Collection

Two individuals (ZQ and ZZ) extracted study data independently. There was no significant difference of opinion, and the third review authority did not need to arbitrate the data extraction. Two review authors (WT and HL) verified and input the data, and both of them confirmed the studies' eligibility. A summary table including study author(s), publication date, number of people in the study, medication, and dosage information of the experimental group and placebo group, treatment duration, and results was prepared (Table 1). We tried to contact the authors for more original information if there were incomplete data. We calculated summary statistics such as mean and SD and then entered databases on the complete datasets provided by trial authors. One of the included studies was excluded because the author could not be contacted and the data could not be extracted.

**Table 1:** Characteristic of 10 studies included in the meta-analysis.

**Study, year (country)**	**Study detail**	***n***	**Age**	**Experimental group**	**Control/placebo group**	**Duration**	**Outcome summary**
Majamaa et al., (28); Finland	Randomized double-blind study	27	2.5–15.7 months	EHF with LGG (5 × 10^8^ cfu/gm)	EHF only	Two months	SCORAD improved significantly during the 1-month study period in infants treated with the EHCF with Lactobacillus GG.
Viljanen et al., (29); Finland	Double-blind placebo-controlled	38	1.4–11.9 months	EHF with LGG (ATCC 53,103) LGG (5 × 10^9^ cfu/gm formula, twice daily)	EHF with microcrystalline cellulose	Four weeks	4 week's treatment with LGG may alleviate intestinal inflammation in infants with CMA.
Viljanen et al., (30); Finland	Double-blind placebo-controlled trial	78	1.4–11.9 months	EHF with LGG (ATCC 53,103) LGG (5 × 10^9^ cfu/gm formula, twice daily)	EHF with microcrystalline cellulose	Four weeks	Probiotics did not improve scord in a large group of infants with CMA.
Baldassarre et al., (31); America	Prospective, randomized, double-blind, placebo-controlled study	26	Mean age of 4.03 months	EHCF with LGG (2.50 × 10^7^-5 × 10^8^ cfu/gm)	EHCF only	Four weeks	EHCF + LGG resulted in significant improvement of hematochezia compared with the EHCF alone.
Berni Canani et al., (32); Italy	Randomized controlled open trial	55	1–12 months	EHCF with LGG (at least 1.4 × 10^7^ cfu/100 ml)	EHCF only	Twelve months	Diet based on LGG supplementation of EHCF may reduce the time of tolerance acquisitions of CMA.
Berni Canani et al., (22); Italy	Open nonrandomized trial	119	<12 months	EHCF with LGG (dose not given)	EHCF only	Twelve months	EHCF+LGG accelerates tolerance acquisition in children with CMA.
Berni Canani et al., (33); Italy	Open randomized trial	19	1–12 months	EHCF with LGG (4.5 × 10^7^-8.5 × 10^7^cfu/gm)	EHCF only	Twelve months	EHCF+LGG promotes tolerance in infants with CMA.
Basturk et al., (34); Turkey	Randomized double-blind placebo-controlled trial	106	Mean age was 68.75 ± 5.32 days and 66.4 ± 4.36 days in probiotic and placebo groups, respectively	LGG 10^9^ cfu and corn oil, at a dose of five drops a day orally for 4 weeks	Dietary with placebo with-out LGG	Four weeks	Receiving dietary LGG with cow's milk-free diet significant improvement in symptoms of infants diagnosed CMPA.
Paparo et al., (35); Italy	Randomized controlled trial	20	Mean age was 6.0–8.0 months and 6.0–9.0 in probiotic and placebo groups, respectively	EHCF with LGG (dose not given)	Soy formula	Twelve months	Dietary intervention could exert a different epigenetic modulation on the immune system in CMA children.
Rita Nocerino et al., (36); Italy	A prospective cohort study	365	5 months	EHCF + LGG (dose not given)	Rice hydrolyzed formula, soy formula, EHWF, or amino acid–based formula	Three years	EHCF + LGG can accelerate the time to gain immune tolerance.

#### Statistical Analysis

Three authors (WL, ZQ, and ZZ) analyzed and processed the data using the RevMan 5.3 software (http://community.cochrane.org/tools/review-production-tools/revman-5/). We chose the SCORAD index to evaluate the effectiveness of probiotics. Since the results are expressed as continuous data, the mean difference (MD) and 95% confidence interval (CI) were used for statistical analysis. If the data obtained were dichotomous data, such as the number of people who have milk tolerance, the risk ratio and 95% CI were used to present the results. Moreover, we chose the random-effect or fixed-effect mode according to the heterogeneity. Besides, we also used the chi-square test to identify statistical heterogeneity. The I^2^ statistic was used to identify and quantify heterogeneity, where I^2^ ≥ 50% indicated an obvious heterogeneity; if not, then there was no heterogeneity. For significant heterogeneity, we used a random-effect model; for no heterogeneity, we employed the fixed-effect model. Statistical significance was set; two-tailed *p* < 0.05 was used to reflect statistical significance.

We followed the PRISMA guideline (http://www.prisma-statement.org/) and used its flowchart to show the specific process of study screening and exclusion. It also showed the relevant meta-analysis consequences through the forest diagram. Moreover, by using the “risk of risk” tool in RevMan software, we did find some risks of bias in all studies. Due to the small number of literatures during the meta-analysis, no assessment of publication bias was performed.

#### Quality Assessment of Research Results

The “Grades of Recommendation, Assessment, Development, and Evaluation” (GRADE) approach provides guidance for the rating of the quality of evidence and the strength of recommendation in healthcare. It is of great significance for systematic review and summary of evidence. We applied the GRADE approach to score the main comparison results of this study, and to judge the level of evidence quality. We evaluated our main results (CMA tolerance rate) and secondary results (improvement of bowel symptoms and changes in SCORD score after treatment) in accordance with GRADE guidelines (37).

## Results

### Included Studies

The PRISMA flowchart shows our screening process and the reasons for exclusion (Figure 1). We preliminarily screened 953 articles and eliminated nonconforming articles according to the inclusion and exclusion criteria. After screening, only 11 studies were retained. As some studies did not express their data as mean ± SD or specific number of patients, we contacted the corresponding authors by e-mail. However, we did not receive any reply from one author, which resulted in the exclusion of one study. Finally, only 10 studies involving 853 children met our inclusion criteria and eight of them were included in a meta-analysis (Table 1).

### Quality Assessment

**Figure 2:**
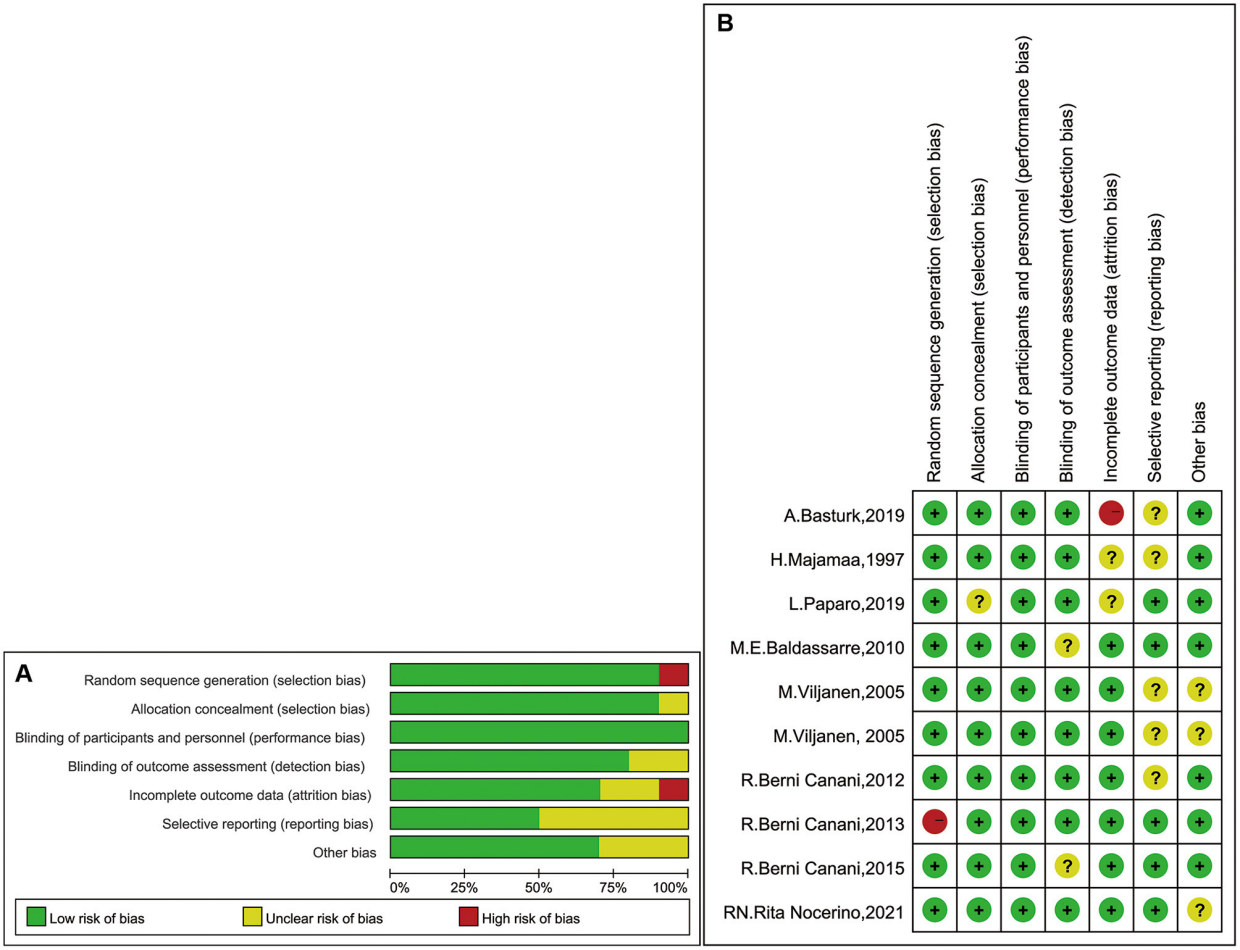
**(A)** Judgments of bias risk graph review authors in the term of each one. Expressed as percentages. **(B)** Judgments of bias risk summary review authors for individual risks in studies.

Figure 2 presents the risk of bias of all enrolled studies and the individual bias risks, adjudged by two authors (WT and HL). Seven studies divided the children into LGG intervention and placebo groups [two trial designs, three groups (LGG, mixed probiotics, and placebo) and one trial design, five groups (LGG, rice hydrolyzed formula, soy formula, EHWF, and amino acid–based formula)]. Seven studies were double-blind trials, and one study was a prospective cohort study. All studies had baseline data such as mean age and growth state. There was no significant difference in baseline data between these groups.

For nine studies, we found that the randomization method had a low risk of bias (28–36). However, in one study, the method used to generate the randomization sequence had a high risk of bias, because the article clearly stated that it was a nonrandomized trial (22). The authors of two studies did not describe the concealment of treatment (32, 35), and two studies did not provide clarification or information on blinding (31, 36). Moreover, five studies provided either unclear data or data presented partially as figures (28–30, 32, 34). Thus, they were classified as unclear risk. In the incomplete outcome data domain, we assessed one study with a high risk of bias due to the large number of people lost to follow-up (34).

### *Lactobacillus rhamnosus* GG and Tolerance of Children With CMA

**Figure 3:**
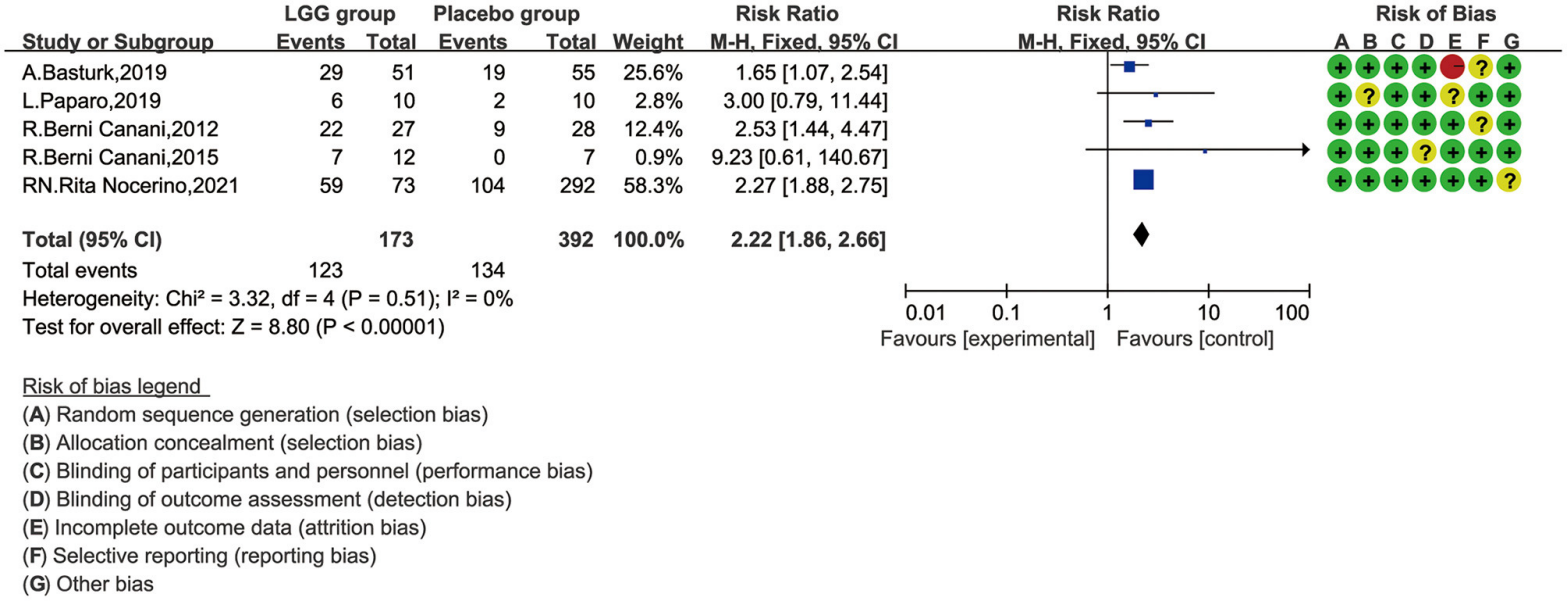
Risk ratio (RR) with 95% confidence interval (CI) and *L. rhamnosus* GG treatment, compared with control and placebo interventions.

Data from 565 children (LGG group, 173; control group, 392) were evaluated (32–36). These five studies have reported tolerance rates and pooled data. One of the studies ended after 36 months of follow-up, while the other four studies ended after 12 months. One study also reported tolerance, but the data could not be used for quantitative synthetic analysis and were excluded (22). This study reported that the tolerance rate of the EHCF + LGG group was 78.9% after follow-up to 12 months, which was significantly higher than that of the non-LGG group; at the same time, binary regression analysis suggests that LGG can enhance the acquisition of tolerance (B 3.35, OR 28.62, 95% CI 8.72–93.93; *p* < 0.001). In order to avoid data loss, we quantitatively synthesized the number of immune tolerances obtained at the end point of follow-up. The results of a meta-analysis of fixed-effect models involving five trials are shown in Figure 3. Significant differences between the tolerance rate of the LGG and control groups were observed overall, and the results showed that LGG was more able to gain immune tolerance (RR, 2.22; 95% CI, 1.86–2.66). Meanwhile, no obvious heterogeneity was found in these five trials (*I*^2^ = 0.0%, *p* < 0.00001).

### *Lactobacillus rhamnosus* GG and SCORAD Value of Children With CMA

**Figure 4:**
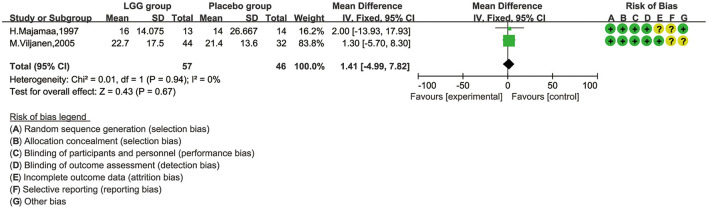
The comparison of mean difference (MD) scoring and SCORAD values between *L. rhamnosus* GG control and placebo interventions.

There were only two studies, involving 103 children, that reported SCORAD (28, 30). Majamaa reported that the SCORAD score improved significantly after 1 month of intervention in patients who received LGG (*p* = 0.008), but not in those without LGG (*p* = 0.89). However, in IgE-mediated CMA infants, the decline in SCORAD in the LGG group was greater than that in the placebo group, from baseline to 4 weeks after treatment (26.1 vs. 19.8, *p* < 0.036); Viljanen found that there was no significant difference in the changes in the SCORD scores before and after treatment between the LGG group and the control group. For meta-analysis, we chose the fixed-effect model and found that there was no obvious heterogeneity, either (Figure 4). Additionally, we did not find any significant difference [mean difference (MD), 1.41; 95% CI, −4.99–7.82; *p* = 0.67].

### *Lactobacillus rhamnosus* GG and Symptom Improvement of Children With CMA

Two trials involving 136 patients reported changes in allergic children with CMA (28, 31). Baldassarre conducted a study to explore whether LGG can aid the recovery of infants with CMA. The author found that the fecal occult blood in the LGG group was significantly improved. There was no fecal occult blood in the LGG group after the 4-week follow-up, while 5/14 cases in the control group were positive for fecal occult blood (*p* < 0.002). Meanwhile, compared with the placebo group, the average decrease in fecal calprotectin in the LGG group was greater (−14.5 ± 107.93 vs. −112.7 ± 105.27 mg/g, *t* = 2.43, *P* = 0.02). Basturk drew similarly optimistic conclusions. The study claimed that the intestinal symptoms of the probiotic group were significantly improved compared to the placebo group (*p* < 0.001). However, there was no significant difference in the complete recovery rate between the two groups, although the recovery rate of the probiotic group was higher (62 vs. 37%, *p* = 0.147).

**Figure 5:**
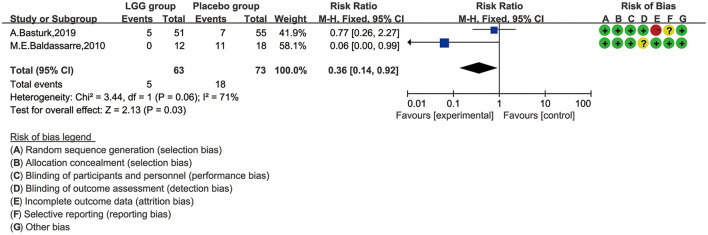
Risk ratio (RR) scoring with *L. rhamnosus* GG treatment, compared by fecal occult blood test and with control and placebo interventions.

We selected the negative rate of the fecal occult blood test for quantitative synthesis of data. We tried to use the random-effect model for data analysis. Heterogeneity was found (*I*^2^ = 71%), and the random-effect model was finally selected. As shown in Figure 5, the LGG group can obtain a higher negative rate of fecal occult blood test compared with the placebo group, and there was a significant difference between the two groups (RR, 0.36; 95% CI, 0.14–0.92; *p* = 0.03).

### *Lactobacillus rhamnosus* GG and Feces Sample of Children With CMA

There were two studies including 64 children that analyzed the fecal sample. However, because the data were not presented as mean ± SD, no quantitative synthesis was performed.

One of two studies showed that the concentration of AT was significantly decreased after 1 month of LGG treatment (*p* = 0.03), but it was not statistically significant in the control group (*p* = 0.68) (28). Moreover, this study found that the LGG treatment could reduce the concentration of TNF-α in fecal samples, and the difference was statistically significant (*p* = 0.003). However, during the trial, we did not find any significant differences in the concentration of ECP between the LGG group and the placebo group.

Another study suggested that TNF-α showed no difference between the LGG treatment group and the placebo group (29). However, in children with IgE-mediated CMA, TNF-α in the LGG group tended to be less than that in the placebo group after being exposed to a milk challenge. Moreover, the fecal ECP did not respond to treatment. The study showed that infants with IgE-mediated CMA had a greater tendency to have increased AT after milk exposure than the control group.

### *Lactobacillus rhamnosus* GG and Adverse Events of Children With CMA

In this meta-analysis, there were no included trials that reported adverse events of LGG in children with CMA.

### Quality of the Evidence (GRADE)

**Figure 6:**
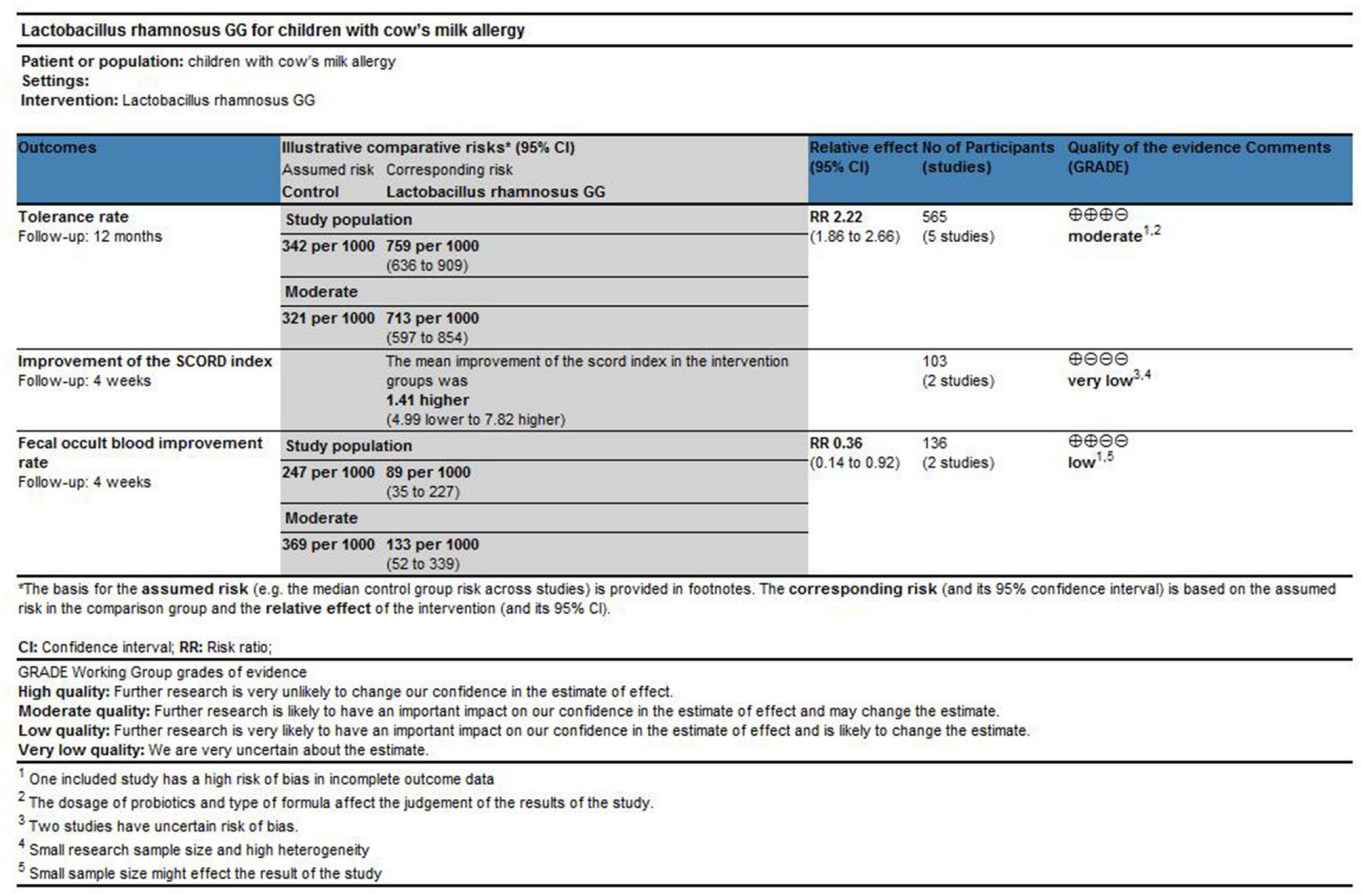
Quality assessment of the main results of the study by the GRADE tools.

As shown in Figure 6, we used the GRADE tool to evaluate the main outcomes of this study. For the effect of LGG on the tolerance rate of CMA in children, we downgraded the quality of evidence to moderate because the included studies had a high risk of bias and confounding factors may affect the judgment of the results. For the improvement effect of LGG on intestinal symptoms in children with CMA (measured as “fecal occult blood test positive rate”; binary outcomes), we downgraded the quality to low because of the small number of studies reporting this outcome and the high risk of bias. For the improvement of CMA symptoms at the end of treatment (measured using the SCORAD index; continuous outcomes), we downgraded the quality to very low because the number of studies that reported this result is small and the quantitative analysis results suggest significant heterogeneity.

## Discussion

Overall, the data showed some benefit of LGG supplementation in children with CMA; children using LGG can achieve immune tolerance more easily. We investigated the tolerance rate of children with CMA after using LGG for 12 or 36 months and determined the use of LGG can help achieve better immune tolerance (RR, 2.22; 95% CI, 1.86–2.66; moderate-quality evidence). The secondary results demonstrated the impact of LGG treatment on the SCORAD value and the change in the negative rate of occult blood in stool specimens. The results of the meta-analysis indicated that there was no significant difference in the reduction of SCORD scores before and after treatment between the two groups. However, the analysis of stool samples showed that LGG can alleviate the intestinal symptoms of children with CMA and improve fecal occult blood. Simultaneously, LGG has no effect on the concentrations of TNF-α, AT, or ECP. These indicators are involved in immune function and inflammation. However, we need to be cautious about the conclusions of this study, because some studies include only limited infant data.

Reduced oral tolerance is the cause of food allergy, and oral tolerance is a gut-associated lymphoid tissue antigen uptake default immune response (38). Changes in the number or diversity of intestinal microbes may affect oral tolerance and make people susceptible to food allergies (39). Several studies have shown that early colonization of gut microbes can affect the development of atopic diseases (40, 41).

As the double-blind RCT showed, researchers injected probiotics into pregnant women and into their infants who had high-risk factors for atopic diseases, for 6 months postnatally, and evaluated the probiotics' preventive effect (42). The results showed that the incidence of specific diseases in the probiotics group was significantly lower than that in the placebo group [23 vs. 46%; RR = 0.51 (95% CI 0.32–0.84)], and probiotics could prevent early atopic disease in high-risk children. However, other RCTs on the role of probiotics against allergic disease showed that the use of probiotics had no significant effect on the incidence of allergic disease by age 2 years (43). Meanwhile, according to the GRADE evidence to decision frameworks, the WAO guideline panel made two recommendations based on the low accuracy of the evidence, namely, the use of prebiotic supplementation in breastfed babies and the need for prebiotic supplementation in non-breastfed infants (44).

LGG might play its role in the intestinal protection through the regulation of the host immune response and exertion of specific local and systemic effects (19). A study of children's diarrhea in India showed that LGG has a positive immunomodulatory effect and can improve intestinal permeability (45). However, Cabana et al. conducted an RCT and noted that early LGG supplementation has no effect on preventing eczema in 2-year-old children (43). Furthermore, it showed that, at 2 years of follow-up, the estimated cumulative incidence of eczema in the placebo group was 30.9% (95% CI, 21.4–40.4%), compared with 28.7% (95% CI, 19.4–38.0%) in the LGG group, and the hazard ratio was 0.95 (95% CI, 0.59–1.53) (log-rank *p* = 0.83). An intervention meta-analysis on probiotics also showed that LGG did not have effects on atopic dermatitis (46); however, the subjects were not children with CMA. Moreover, we found that LGG can promote oral tolerance in children (RR, 2.22; 95% CI, 1.86–2.66; moderate-quality evidence) and demonstrated that it can alleviate the symptoms of intestinal occult blood, but the effect is weak (RR, 0.36; 95% CI, 0.14–0.92; low-quality evidence). Furthermore, some cytokines related to inflammation or immunity (TNF-α, AT) changed significantly. Our results were consistent with those of Guest et al., who concluded that CMA infants fed with LGG formula milk powder were more likely to acquire milk tolerance (47). However, this study did not consider the suitability of using different formula milk powders, and the influence of confounding factors including comorbidities or underlying diseases cannot be ruled out. The RCT studies we included had good population homogeneity, which fully excludes the impact of potential diseases on the results. Moreover, our results suggested that LGG had no effect on the SCORAD values (MD, 1.41; 95% CI, −4.99–7.82; very low-quality evidence) or inflammation as well as immunological indicators. This could be due to the small number of included trials. Thus, we contacted the corresponding author for more data, but to no avail. Therefore, we excluded some studies, which may directly affect our conclusions. Thus, our secondary results appeared statistically nonsignificant. These topics require further attention. TNF-α is a marker of increased intestinal permeability (48). An animal study showed that LGG can inhibit the *in vitro* secretion of TNF-a that activates murine macrophages (49). More research to explore the influence of LGG on inflammatory markers is warranted.

As far as we know, there are limited quantitative studies on the effectiveness of LGG on children with CMA. In our meta-analysis, only two studies used the SCORAD method to assess the effectiveness of the LGG treatment. One trial showed no obvious effect (30), while another suggested that LGG is beneficial for improving the symptoms of CMA (28). The inconsistency of this result may be related to the difference in intervention time and sample size; further experiments are needed to confirm this.

One study revealed no serious adverse events associated with LGG use in low-birth-weight infants, indicating the safety of LGG (50). Several cases of adverse reactions to the use of LGG have been reported. For example, some children with short-bowel syndrome seem to suffer from sepsis with LGG-like bacteria after treatment with LGG (51, 52) and an extremely preterm infant was diagnosed with a catheter-related infection caused by the oral LGG (53). There were no adverse events analyzed in our review, and it is necessary for the investigator to expand the sampling size while strengthening the corresponding follow-up or extending the follow-up time.

This study has certain advantages. Unlike other meta-analyses of the same type, we only included and analyzed the RCT study of LGG, so we included more specific types of probiotics as the research object. This can give future researchers a clearer research direction. Additionally, the included studies were RCTs, and the conclusions of these studies were relatively rigorous and scientific. However, our meta-analysis had some certain limitations. First, we excluded some RCTs from this meta-analysis, and the small number of included studies could have reduced the reliability of the data and increased the heterogeneity and reporting bias. Furthermore, although the heterogeneity of the main results was small, we did not perform a subgroup analysis and sensitivity analyses due to the small sample size, which may have concealed the possible bias. Finally, we did not capture the adverse events that occurred in the application of LGG in children with CMA, which also requires us to be cautious about the conclusions of this study.

## Conclusion

Our results indicated that LGG may have moderate-quality evidence to promote oral tolerance in children with CMA, and it may have a role in promoting the recovery of intestinal symptoms. However, this conclusion must be treated with caution due to the small number of included studies. Although this result shows that the management of children with CMA has a positive trend, more powerful RCTs with standardized measurements are needed to evaluate the most effective dose and treatment time for children with CMA and fully understand its potential adverse reactions.

## Data Availability Statement

The original contributions presented in the study are included in the article/supplementary material, further inquiries can be directed to the corresponding author.

## Author Contributions

WL and WT proposed this research idea and conducted the research design and interpreted the statistical results. WL, WT, and HL carried out the research screening and quality assessment. ZQ and ZZ conducted the data screening and extraction. WL, ZQ, and ZZ used RevMan software to evaluate bias and perform statistical analysis. WT drafted the full text of the original manuscript. WL critically reviewed and revised the manuscript. All authors contributed to the article and approved the submitted version.

## Conflict of Interest

The authors declare that the research was conducted in the absence of any commercial or financial relationships that could be construed as a potential conflict of interest.

## Publisher's Note

All claims expressed in this article are solely those of the authors and do not necessarily represent those of their affiliated organizations, or those of the publisher, the editors and the reviewers. Any product that may be evaluated in this article, or claim that may be made by its manufacturer, is not guaranteed or endorsed by the publisher.
